# Understanding speech in “noise” or free energy minimization in the soundscapes of the anthropocene

**DOI:** 10.3389/fnins.2025.1534425

**Published:** 2025-03-14

**Authors:** Daniel J. Strauss, Alexander L. Francis, Zeinab Schäfer, Matthias Latzel, Farah I. Corona–Strauss, Stefan Launer

**Affiliations:** ^1^Systems Neuroscience and Neurotechnology Unit, Faculty of Medicine, Saarland University, and School of Engineering, htw saar, Homburg/Saar, Germany; ^2^Speech Perception and Cognitive Effort Lab, Department of Speech, Language and Hearing Sciences, Purdue University, West Lafayette, IN, United States; ^3^Department of Computer Science, Aarhus University, Aarhus, Denmark; ^4^Sonova AG, Stäfa, Switzerland

**Keywords:** hearing, evolution, noise, free energy principle, attention, listening effort, speech

## Abstract

Listening to speech in the presence of irrelevant sounds is ubiquitous in the modern world, but is generally acknowledged to be both effortful and unpleasant. Here we argue that this problem arises largely in circumstances that our human auditory system has not evolved to accommodate. The soundscapes of the Anthropocene are frequently characterized by an overabundance of sound sources, the vast majority of which are functionally irrelevant to a given listener. The problem of listening to speech in such environments must be solved by an auditory system that is not optimized for this task. Building on our previous work linking attention to effortful listening and incorporating an active inference approach, we argue that the answers to these questions have implications not just for the study of human audition. They are also significant for the development and broad awareness of hearing aids and cochlear implants, as well as other auditory technologies such as earbuds, immersive auditory environments, and systems for human-machine interaction.

## 1 Introduction

The Anthropocene, a term referring to our current era within the geological epoch of the Holocene, is marked by human activities that radically alter our soundscapes, see Habib et al. (2007); Swaddle et al. (2015); Slabbekoorn (2018). Modern soundscapes incorporate sounds produced by large numbers of people, traffic, machinery, phones, radios, televisions, etc. but also the indirect sounds produced by reverberations from installed man-made objects and infrastructure. In the acoustically rich and complex soundscape of the Anthropocene, the amount and variety of unimportant and unwanted sound subsumed as ”noise” has steadily increased (Habib et al., 2007; Slabbekoorn, 2018).

The development of transportation systems, encompassing vehicles, aircraft, and maritime vessels, has extended noise pollution into previously remote areas. Industrialization, through activities such as mining and energy production, further exacerbates noise pollution. These drastic changes in the soundscape clearly impact wildlife but also human habitats (Habib et al., 2007; Swaddle et al., 2015; Slabbekoorn, 2018). Similarly, anthropogenic climate change has indirect effects on natural soundscapes by altering weather patterns, habitats, and animal behavior, but also reshapes regional sound compositions critical to human wellbeing (Lorenzi et al., 2023).

Overall, the Anthropocene has ushered in a notable escalation in human-induced noise pollution, necessitating concerted efforts in sound monitoring, regulatory measures, and the adoption of quieter technologies to mitigate its adverse effects on ecosystems, wildlife, and human health.

With respect to anthropogenic changes that directly affect human listening, the most significant of these is urbanization, which has introduced heightened levels of human-generated sounds, and concentrated humans together in unprecedented numbers.

Thus, there has been a change in the soundscape, but also in what we need to get from the soundscape. In earlier eras such as the Pleistocene epoch, the time period in which modern humans evolved (Tooby and Cosmides, 1992), listening to the non-human world was much more important for survival, for example in order to avoid threats and achieve goals (hunting, gathering, etc.). Sudden and/or high-intensity, ”attention-grabbing” sounds were likely to be important for survival, potentially signaling significant changes in the immediate environment, and thus early auditory systems (i.e., those inherited by early hominins) would likely have already evolved to treat them with priority. Surrounding speech was likely produced by known individuals, and was likely to be important for social interaction and, ultimately, survival. In the Anthropocene, we use our senses in a very different way than in the deep past. We are (mostly) not threatened by anything that makes sound but is not human. A sudden sound like the breaking of a nearby branch, or the warning call of a bird or small mammal, is functionally irrelevant to most humans in the Anthropocene. Even the sound of a neighbor's car door slamming, or the clatter of glasses in a restaurant kitchen, while attention-demanding, is generally functionally irrelevant. The relative significance of exogenously-directed auditory attention has changed radically in many modern contexts, typically with far less relevance to immediate survival except, quite notably, in the case of avoiding traffic. And yet, despite the decline in the fitness benefit of orienting toward sudden, loud, warning-like sounds, there are ever more sounds in the environment that may cause an involuntary switch of attention—for instance, the squeal of a tram, the slam of a door, a car horn. Even though none of these sounds may be relevant, they all exhibit acoustic properties that make them attentionally demanding, i.e., distracting, and the repeated capture of attention by irrelevant sounds quickly becomes annoying and stressful.

Using careful but extremely incomplete phylogenetic information from Ackermann et al. (2014); Gintis (2011); Chen and Wiens (2020), we have loosely sketched the changing complexity of soundscapes in urbanized areas (see also Slabbekoorn, 2018) and auditory capacity in a conceptual time relation. The point is to show that evolutionary timescales and the timescale of changing soundscapes of the Anthropocene differ vividly. Here, we are deliberately vague as to what exactly constitutes auditory capacity, though for the present, we can roughly define it as the ability for sound processing and production in acoustic communication in vertebrates (in the Mesozoic Era) and our lineage in primates later in the Cenozoic Era. In primates, sophisticated vocal learning, speech, and comprehensive musicality seem to be specific to humans (*Homo sapiens*) (Dichter et al., 2018; Aboitiz, 2018; Patel, 2021), though some aspects of spoken language were likely present in other now-extinct *Homo* species, including *neanderthalis* (Conde-Valverde et al., 2021) and *erectus* (Swedell and Plummer, 2019; Everett, 2017). In fact, auditory capacity has not changed much since Homo sapiens appeared (approx. 300,000–200,000 years ago), see Everett (2017). For a more general review on the development of acoustic communication and auditory capacity within mammals, we refer to Grothe et al. (2004); Sterbing-D'Angelo (2009); Ackermann et al. (2014); Manley (2017); Chen and Wiens (2020).

**Figure 1:**
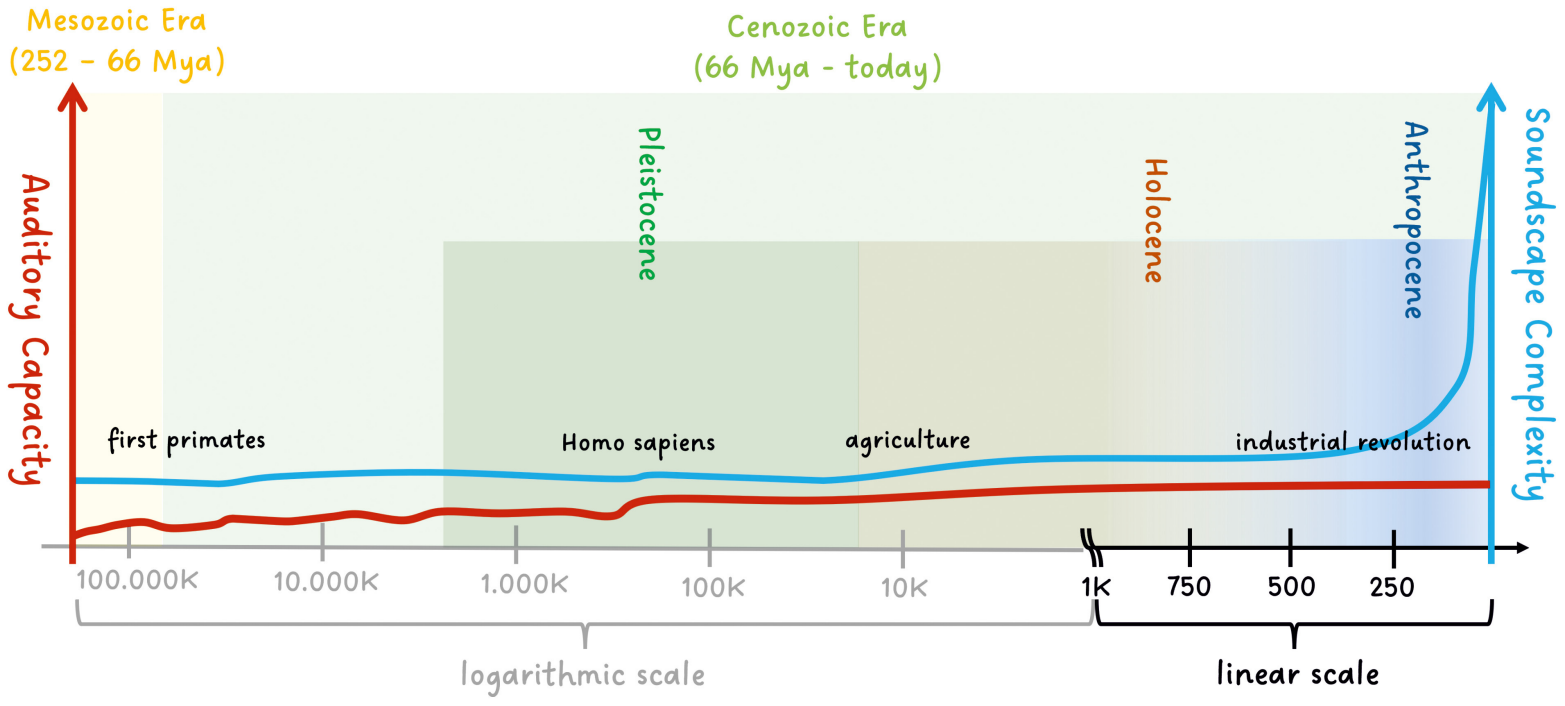
Conceptual sketch of the changing complexity of soundscapes and the auditory capacity in the Mesozoic and Cenozoic Era. Whereas the auditory capacity is rather constant since the Homo sapiens appeared, the soundscapes of urbanized areas changed drastically since the industrial and technological revolution.

Since the arrival of Homo sapiens, beside genetic drifts (Star and Spencer, 2013) and epigenetic factors (Ashe and Colot, 2021), cultural evolution has far outpaced any changes due to evolution. Cultural change has altered our environmental soundscapes in urbanized areas drastically, in particular since the industrial revolution and the exponentially increasing use of technology, see Figure 1. Note that this discussion does not include the acceleration of human adaptive evolution due to gene-environment interactions and a gene-culture co-evolution (e.g., see Hawks et al., 2007; Gintis, 2011) or factors which are not directly related to the “auditory capacity”. It is also worth emphasizing that, in the following section, we focus on deeply anchored auditory attention mechanisms in Homo sapiens and not on culturally dependent learning and adaptation mechanisms of the attention system within our species (Boduroglu and Shah, 2017; Jurkat et al., 2020) in the modern age.

## 2 Conceptual model for understanding speech in noise

So how do attention mechanisms in hearing from the times of hunters and gatherers fit the modern world? Listening occurs in a soundscape that is always present. Outside the hearing clinic or laboratory it is virtually impossible to find a single acoustic event occurring in isolation. The ability to subset sensory space to better distinguish relevant from irrelevant phenomena is ecologically essential (Stevens, 2013; Lev-Ari et al., 2022; Bruner and Colom, 2022), and plays a particularly important role in social primates (Schülke et al., 2020). However, unlike in vision, humans and apes do not significantly move their pinnae toward sounds, even though we retained a vestigial pinna-orienting system that has persisted as a “neural fossil” within in the brain for about 25 million years (Hackley, 2015; Strauss et al., 2020). So, we cannot move our ears as we can move our eyes to focus on a part of the visual scene. We do not have an auditory fovea, at least not in a physical sense. Thus in listening, unlike in vision, the neural representation of the physical world remains unchanged by the peripheral sensor itself. In order to tune in to a particular auditory object within a soundscape (auditory scene) we need to use attention (Shinn-Cunningham, 2008).

Here, to distinguish between the target sound and the unwanted sound, i.e., the noise, we can employ the binary figure (target) and background (noise) principle from vision, see Marr (1982). The idea of figure and background is very important because there is always a whole soundscape. What distinguishes figure from ground are the “goals” of the listener. Note that, although typically such “goals” are considered in terms of explicit representations (e.g. “I want to listen to that person, not this one”), we prefer to consider them more broadly, even including such “corporeal beliefs” as “I want to avoid danger” (see Parvizi-Wayne, 2024). Thus, a voice might shift from background to foreground either because I choose to attend to it, or because it has become louder and higher pitched, as it might if the speaker is angry and potentially becoming a threat.

In the pre-Anthropocene era, many sounds in the soundscape were crucial for survival, and natural selection would over time have tuned our senses, and our attention, to better respond to them. In the sense of Parvizi-Wayne (2024), rapid response to sudden, loud sounds, for example, should be deeply entrenched in the predictive model that guides what we pay attention to. Thus, an animal hunting or foraging in a small group might be listening primarily to the environment, highly sensitive to any change that might signal the approach of a threat or loss of an opportunity. In species that forage in groups, including both baboons and chimpanzees, this includes listening not just to the sounds of the environment, but also to one's friends and neighbors, who are often both allies and potential rivals. That is, although we often focus on the survival benefits of listening in the context of predator/prey interactions, it seems likely that, in a social animal the ability to listen to relevant communication (directed both to the listener and to others) is at least equally significant (Schülke et al., 2020).

### 2.1 Segregating the target from “noise”

**Figure 2:**
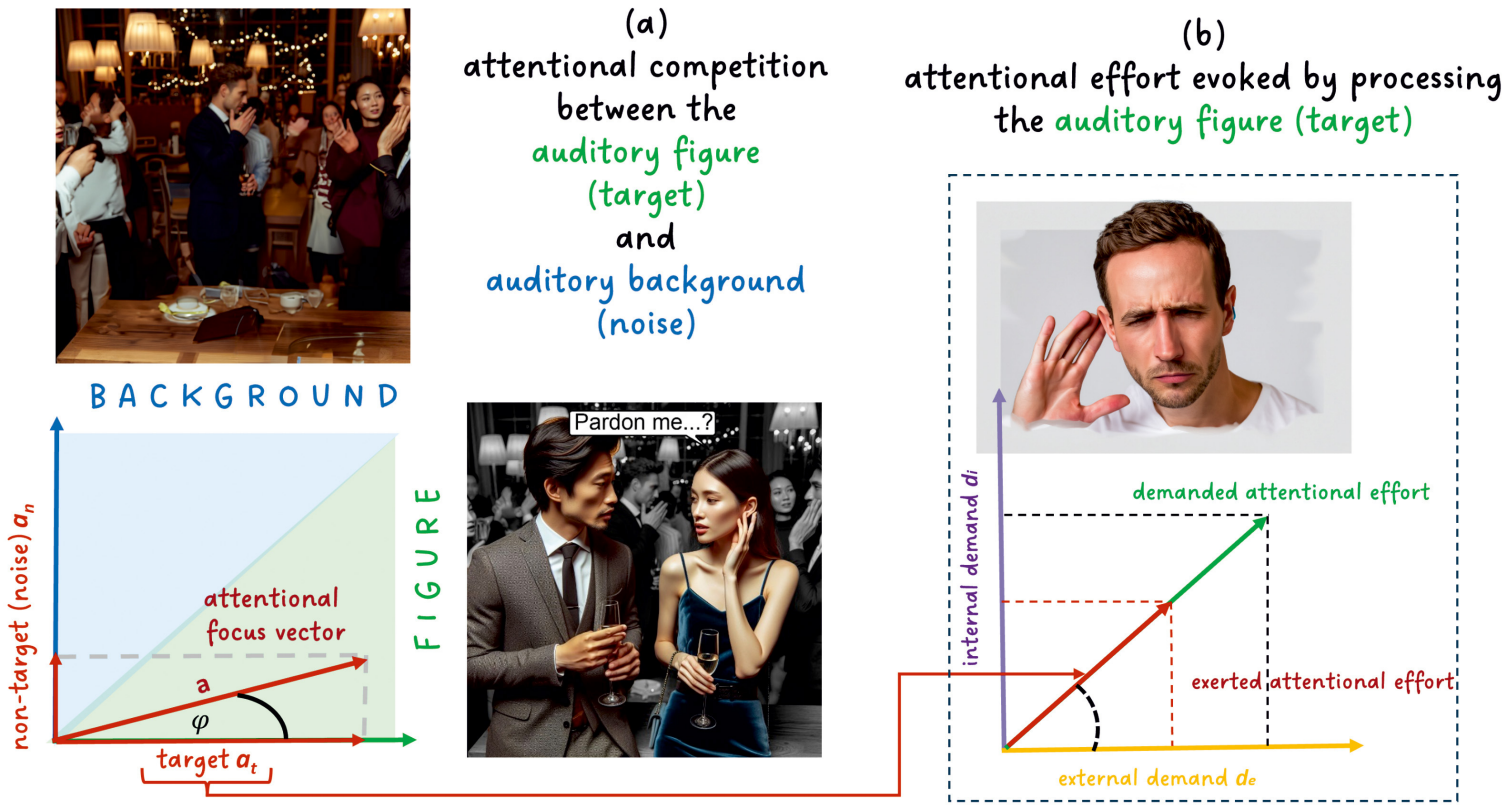
The relation between attention and effort in listening: **(A)** Model of the attentional competition between auditory target and background based on a modified version of the two-competitor model in Strauss et al. (2024b). It is a dynamical model in which effort varies in the 2D plane, and we have to invest effort to keep the attentional focus vector on the target. **(B)** The associated effort of the target processing when employing the taxonomic model of attention in effortful listening in Strauss and Francis (2017). Here, the exerted attentional effort (Sarter et al., 2006; Strauss and Francis, 2017) is driven by the internal demand d_*i*_ (e.g., requiring attention to working memory objects to make sense of complicated spoken sentences) and the external demand *d*_*e*_ (e.g., requiring attention to perform stream segregation to separate the target from the “noise”), see Strauss and Francis (2017); Strauss et al. (2024b) for details.

There is a strong link between different modes of attention and effortful listening to speech as a target in noise (see Strauss and Francis, 2017). Typically, one starts with the classic taxonomy of exogenous attention (bottom-up, automatic, unconscious) and endogenous attention (voluntary/top-down, goal-directed) in sensory processing (see Müller and Rabbitt, 1989; Jigo et al., 2021; Ren et al., 2021) frequently applied in auditory scene analysis (Bregman, 1990). However, understanding the distribution of attention does not necessarily depend on a strict division of these concepts. For example, following Parvizi-Wayne (2024), the exogenous attraction of attention by an external stimulus (e.g., a sudden, loud sound) can still be understood as the deployment of attention toward a stimulus on the basis of goals, albeit goals that may be deeply entrenched in the predictive, hierarchically organized model of the environment (e.g., “Identify the source of sudden, loud sounds”) as a result of millennia of natural selection. Thus, for example, attentional focus can be modeled by a continuous (probabilistic) stream selection model depending on weights related to exogenous and endogenous processes (Trenado et al., 2009; Strauss et al., 2010) or by a single-agent model when using active inference, which does not need a dichotomy of these attention concepts (see Parvizi-Wayne, 2024 and below). In either case, the probabilistic selection scheme in Trenado et al. (2009) is akin to the biased competition model in visual perception (Desimone and Duncan, 1995); see also Shinn-Cunningham (2008) for an adaptation of biased competition to the auditory modality and Strauss et al. (2010) for a mapping to effortful listening. The two-competitor as well as taxonomic models of attention in effortful listening are summarized in Figure 2, showing a typical cocktail party situation where two people are having a conversation (target) in a noisy background (see also below).

The predictive (generative) hierarchically organized model of the environment employed in Feldman and Friston (2010); Clark (2013); Parvizi-Wayne (2024) might, for the sake of simplicity in structure, be mathematically seen as a generative spatiotemporal scale space, i.e., a nested structure encompassing multiple spatiotemporal scales of the (predicted) environment. This scale space covers predictions about mostly fast and nearby events at an evolutionarily deeply entrenched core of the model. The further we move away from this core, the more complex and forward-thinking (in time and space) these predictions might be. Building on Feldman and Friston (2010) that treats biased competition in terms of an active-inference framework, Parvizi-Wayne (2024) draws on Friston's free energy minimization theory (Friston, 2010) to represent the often distinct ideas of exogenous and endogenous attention in a unitary, active inference framework. As this framework comprehensively supports a mapping of the relation between perception and action in effortful listening, let us take a more formal look at its structure.

### 2.2 Free energy principle in effortful listening

In real-world scenarios such as the cocktail party in Figure 2, listening is not happening in isolation, e.g., deprived of the other senses. Regularities across senses, e.g., integrating lip reading or posture with listening, are crucial for understanding speech in noise, see, e.g., Rosenblum (2008) and also our discussion of relation between perception and action below. However, to avoid an over-generalization, we focus on the auditory modality in the following formal discussion, assuming implicitly that the brain has an internal scale space representation of the entire multisensory environment. We formulate the free energy according to Parr et al. (2022) as follows for our auditory setting; The internal state of the generative scale space model corresponds to a distribution *Q*(*s*), which captures the brain's expectations and prior beliefs about the acoustic scene *s* (including both figure and background across spatiotemporal scales). Let *o* represent sensory input, such as the mixture of sounds in a cocktail party scenario. The free energy *F*(*Q, o*) can be expressed as:


$$\begin{array}{l} \underset{\text{free energy}}{\underset{︸}{F\left(Q,o\right)}}=\underset{\text{expectation term}}{\underset{︸}{E_{Q\left(s\right)}\left[-\ln\;p\left(s,o\right)\right]}}-\underset{\text{entropy term}}{\underset{︸}{H\left[Q\left(s\right)\right]}}\geq \underset{\text{surprise}}{\underset{︸}{-\ln\;p\left(o\right)}} \end{array}$$

In this formulation, *Q*(*s*) serves as both the approximate posterior distribution and the internal state itself, representing the brain's model of the acoustic scene. The first term (the negative expected log joint probability, also known as the energy) measures how well the brain's internal model predicts auditory input given its current beliefs. The second term *H*[*Q*(*s*)] which measures the entropy of this distribution, quantifies uncertainty in the brain's beliefs about the acoustic environment. A higher entropy value implies greater uncertainty in the listener's beliefs, making it more receptive to new sensory observations. This adaptability aligns with the Free Energy Principle, which suggests that the brain minimizes free energy by reducing uncertainty and refining its internal model to optimize perception and action in a dynamic environment (Friston, 2010; Parr et al., 2022).

Minimizing free energy requires balancing consistency with the generative model (expectation term) while maintaining appropriate uncertainty through the entropy maximization. In the absence of precise prior beliefs, this formulation follows Jaynes's maximum entropy principle, suggesting the perceptual system should maintain maximum uncertainty about hidden states when information is limited (i.e., higher entropy enables more flexible adjustment of internal models). Importantly, the inequality in the free energy formulation indicates that free energy always provides an upper bound on “surprise”—the unexpectedness of sensory input. By minimizing free energy, the perceptual system indirectly minimizes surprise, making auditory inputs more predictable and allowing for more effective processing of complex acoustic scenes.

This interaction of perception and action might occur quickly and automatically at the evolutionarily deeply entrenched core of the scale space model but also more slowly due to the engagement of more complex and thoughtful schemas as we move away from the core. As predictions and expectations drive attention (e.g., see Grossberg, 2005; Strauss et al., 2010; Clark, 2013; Parvizi-Wayne, 2024), fast and automatic links between perception and action are associated with the “exogenous weights” and slower, reasoning based loops with the “endogenous weights” in the classic terminology (Strauss and Francis, 2017; Strauss et al., 2024b). Parvizi-Wayne's model (Parvizi-Wayne, 2024) emphasizes in this context the importance of “precision weight optimization” over “precision optimization”. This involves not only making predictions across spatiotemporal scales more accurate but also determining the importance (weight) of different pieces of information in their scale space representations (i.e., the multiscale prediction error of perceptual beliefs). If these pieces of information are representations of predicted auditory objects (Shinn-Cunningham, 2008), the following discussion will clarify how this model maps the cocktail party situation illustrated in Figure 2. As we will see, these weights depend on the auditory scene, the context, and the generative model including learned experiences. Precision weight optimization would also map enhanced representations of attended auditory objects along the hearing path due to attention “gain” (or “noise suppression”) neural mechanisms (Strauss et al., 2024a), see Parvizi-Wayne (2024) for more detailed discussions.

Minimizing free energy and surprise directly answers the question of what motivates us to follow a “listening goal”. This “motivational aspect” involves long-term and reasoning-based minimization of (negative) surprises across spatiotemporal scales. For instance, assume the man in Figure 2 is telling the woman what changes are being planned in the dean's office for next week or at the federal level regarding energy prices next winter. As she does not want to encounter surprises in these matters, she is motivated to exert attentional effort in the conversation (Strauss and Francis, 2017). However, free energy minimization also applies to the here and now, providing an almost instantaneous and automatic analysis of sudden loud sounds in the acoustic scene, causing a free energy spike (Parvizi-Wayne, 2024). Consider an abrupt background sound like laughter or clinking glasses at the cocktail party in Figure 2. These automatic processes stem from an evolutionarily deeply entrenched core of the scale space model, securing survival in the present moment. No matter how interesting the conversation about future events is, these acoustically salient events compete for our attention, distract us from the conversation, and cause increased attentional effort to follow the conversation and minimize free energy about future events (called exogenous override in Strauss et al. (2024b)). Here it does not matter that clinking glasses do not need our attention at the cocktail party. As we have stated before, we are not evolutionarily optimized for cocktail parties or other features of modern urban environments. In the modern world, there is an abundance of these free energy spikes caused by sounds addressing the “survival core” of our generative hierarchical model of the environment and also an abundance of acoustic information that might be worth following when minimizing surprises. It is also important to note that attentional shifts in noisy environments can arise from individual priors (i.e., learned experiences) embedded in the brain's internal model, causing one person to focus on a background stimulus linked to past experiences, while another perceives it as irrelevant. For instance, the ringtone of a cell phone might capture more attention if it is the same as one's own, or the sound of a falling tablet could be associated with a threatening situation at a previous cocktail party based on individual experience. Turning to our major theme, the Anthropocene is inducing more free energy in listening situations in multiple ways, vastly increasing the effort of maintaining simple conversations at a cocktail party, let alone in Times Square at rush hour.

## 3 Discussion and technological implications

Technological advances have significantly altered even modern soundscapes within a single human lifetime (Habib et al., 2007; Swaddle et al., 2015; Slabbekoorn, 2018). Cultural evolution, driven by technology, has far outpaced biological evolution (Boyd et al., 2013), leaving us with a sensory processing and perceptual system naturally equipped for environments vastly different from our world today (e.g., see Gazzaley and Rosen, 2016). Our arguments allow us to look at the idea of restoring hearing to its “natural” state from a new angle as our auditory system evolved for vastly different environments. Rather than simply restoring hearing, augmenting hearing through technologies such as noise suppression and directional microphones enables a technological adaptation to Anthropocene soundscapes rather than simply restoring Pleistocene capabilities (see Figure 1 and Tooby and Cosmides, 1992). For the hearing impaired, hearing aids can leverage the hard attentional competition between figure (speech) and background (noise), e.g., by using directional microphones, maybe even informed by physiological signals related to our listening intention (Mikkelsen et al., 2015; Schäfer et al., 2018; Schroeer et al., 2023, 2024). Features such as exogenous cue weighting or dynamic processing modes, informed by evolutionary insights, may improve both safety and user experience in diverse environments (Carretié, 2014; Strauss et al., 2024b; Edwards, 2007). For example, the silent operation of electric cars poses risks by reducing the salience of auditory warning cues (Clendinning, 2018). Artificially adding sound reintroduces a natural correspondence between auditory salience and threat but increases noise pollution (Hegewald et al., 2020; Gilani and Mir, 2021). An alternative is sonifying dynamic traffic information (see ETSI, 2011) via bone-conduction devices, enabling the auditory system to use novel input sources while maintaining its evolved role as a 360° early-warning system (Strauss et al., 2020; Olszanowski et al., 2023). This approach highlights how hearing technologies can integrate evolutionarily honed mechanisms with modern demands. Numerical simulations of conceptual effortful listening models (e.g., see Schneider et al., 2019; Strauss and Francis, 2017) can further contribute to optimizing hearing aid designs, supporting users in navigating modern soundscapes.

Effortful listening arises from mismatches between auditory input and the brain's predictions, linked to increased free energy (Pichora-Fuller et al., 2016; Strauss and Francis, 2017). Predictive models show that internal demands related to a conflict of expectations across spatiotemporal scales and uncertainty drive listening effort, particularly in noisy environments. This framework connects listening effort to broader principles of brain function and allows for experimental exploration of multimodal integration (Calvert et al., 2004; Schulte et al., 2023) in effortful listening. Effortful listening informs the design of acoustic human-machine interfaces, particularly in noise-heavy environments like factories or vehicles (Neumann et al., 2021; Damian et al., 2015; Gonzalez-Trejo et al., 2019). Neuroergonomic approaches (Parasuraman, 2003) can minimize cognitive load by aligning design with the attentional system's evolutionary strengths, creating more intuitive and effective interfaces (Mehta and Parasuraman, 2013). Thus, the computational implementation of the concepts presented in Section 2 (see also Bogacz, 2017; Strauss et al., 2024b) might support the optimization of neuroergonomic designs in medical, human-machine-interaction, and entertainment applications dealing with effortful listening.

## 4 Conclusions

We have considered the understanding of speech in noise within Anthropocene soundscapes from an evolutionary perspective. We propose that there have been, at most, marginal changes in auditory capacities over the last 200,000 years and essentially no changes in the last 2,000 years. However, the soundscape has changed drastically in just the last 200 years. Consequently, we propose that much of the effortful and unpleasant nature of extracting target speech and suppressing background noise stems from our auditory system not being adapted to modern acoustic environments. Using models that link effortful listening to attention, we examined the binary attentional competition between figure and background. We integrated these models into the free energy minimization framework to conceptualize attentional effort in listening. This evolutionary cognitive neuroscience approach and active inference model have implications for studying human audition and developing auditory technologies, including earbuds, hearing aids, immersive environments, and human-machine interaction systems.

## Data Availability

The original contributions presented in the study are included in the article/supplementary material, further inquiries can be directed to the corresponding author.
